# Cytokine Storm in Domestic Pigs Induced by Infection of Virulent African Swine Fever Virus

**DOI:** 10.3389/fvets.2020.601641

**Published:** 2021-01-22

**Authors:** Shuchao Wang, Jingyuan Zhang, Yanyan Zhang, Jinjin Yang, Lidong Wang, Yu Qi, Xun Han, Xintao Zhou, Faming Miao, Teng Chen, Ying Wang, Fei Zhang, Shoufeng Zhang, Rongliang Hu

**Affiliations:** ^1^Institute of Military Veterinary Medicine, Academy of Military Medical Sciences, Key Laboratory of Jilin Province for Zoonosis Prevention and Control, Changchun, China; ^2^College of Life Sciences, University of Ningxia, Yinchuan, China

**Keywords:** African swine fever virus, cytokine storm, domestic pig, proinflammatory cytokine, pathogenesis, chemokine

## Abstract

African swine fever, caused by African swine fever virus (ASFV), is a highly contagious hemorrhagic disease of domestic pigs. The current continent-wide pandemic has persisted for over 10 years, and its economy-devastating effect was highlighted after spreading to China, which possesses half of the world pig industry. So far, development of an effective and safe vaccine has not been finished largely due to the knowledge gaps in pathogenesis and immunology, particularly the role of cytokines in the host's immune response. Therefore, we performed experiments in domestic pigs to analyze the kinetics of representative circulating interferons (IFNs), interleukins (ILs), growth factors, tumor necrosis factors (TNFs), and chemokines induced by infection of type II virulent ASFV SY18. Pigs infected with this Chinese prototypical isolate developed severe clinical manifestations mostly from 3 days post inoculation (dpi) and died from 7 to 8 dpi. Serum analysis revealed a trend of robust and sustained elevation of pro-inflammatory cytokines including TNF-α, IFN-α, IL-1β, IL-6, IL-8, IL-12, IL-18, RANTES (regulated upon activation, normal T cell expressed and secreted), and IFN-γ-induced protein 10 (IP-10) from 3 dpi, but not the anti-inflammatory cytokines IL-10 and transforming growth factor-β (TGF-β). Moreover, secondary drastic increase of the levels of TNF-α, IL-1β, IL-6, and IL-8, as well as elevated IL-10, was observed at the terminal phase of infection. This pattern of cytokine secretion clearly drew an image of a typical cytokine storm characterized by delayed and dysregulated initiation of the secretion of pro-inflammatory cytokine and imbalanced pro- and anti-inflammatory response, which paved a way for further understanding of the molecular basis of ASFV pathogenesis.

## Introduction

African swine fever (ASF), a highly hemorrhagic viral disease of domestic pigs, has been internationally epidemic for nearly 100 years ever since the first report in Africa in 1921 (1, 2). The current pandemic of ASF initiated from the introduction to Republic of Georgia in 2007 (3). Since then, ASF has rapidly spread to other Trans-Caucasian countries, Russia and East Europe, and highlighted its enormous threat to global swine industry by conquering the world's biggest pig producer and consumer, China, in 2018 (4, 5). The causative agent of this devastating disease, African swine fever virus (ASFV), is considered the only DNA arbovirus owing to its capacity to infect several tick species in the genus *Omithodoros* (4). Besides soft ticks, which act as invertebrate competent vectors, ASFV just affects mammalian animals in the *Suidae* family, such as warthogs, wild boars, and domestic pigs. In contrast to the natural reservoir host warthogs, in which the infection of ASFV is basically asymptomatic (6), the domestic pigs and wild boars mostly develop severe clinical manifestations after virus invasion and present a mortality rate up to 100% roughly in 7–10 days post infection (7, 8), despite the fact that attenuated virus strains with low virulence may be isolated over an epidemic period in a certain naive region (9–11).

The virus isolates involved in the present continent-wide outbreak of ASF are largely highly pathogenic (8). Common clinical symptoms of domestic pigs suffering from infection of these strains include sustained high fever, reduced feed intake to anorexia, vomiting, watery to bloody diarrhea, conjunctivitis, respiratory distress, reddened skin, cyanosis, severe depression, huddling, and incoordination (8). Pathologically, systematic hemorrhagic lesions and enlargement can be observed in multiple organs including lymph nodes, spleen, kidneys, lungs, liver, and gastrointestinal tract, accompanied by leukopenia, thrombocytopenia, and macrophage destruction (7, 8). Despite many research efforts and increasing knowledge on the pathogenesis, the basic mechanism how ASFV induces these clinical and pathological signs largely remains to be understood. Several hypotheses have been raised. Among them, early studies focused attention on hemorrhagic lesions, the major acute pathological characteristic, and considered it could be related to virus replication, disseminated intravascular coagulation, and massive destruction of macrophage (8). Moreover, leukopenia, largely attributed to lymphopenia, is regarded as a result of apoptosis in lymphocyte populations induced by immune mediators (1, 7, 8). Nowadays, it is generally accepted that secretion of cytokines by the infected monocyte-macrophage system that is considered as the major target of ASFV and secondarily activated lymphocytes is strongly applied in the abovementioned biological processes (12, 13).

Cytokines play pivotal roles in all aspects of immune responses, orchestrating the innate and adaptive immune response, and therefore, most of the time, act as protectors against both intrinsic and extrinsic nocuous stimuli, such as tissue injuries and microbe invasions (14). However, an exaggerated immune activation with excessive cytokine release could be rather pernicious. An important instance is the so-called cytokine storm, also known as cytokine release syndrome (15). The term “cytokine storm” was firstly coined in the early 1990s to describe the pathological outcome related to organ transplantation when an inflammatory response flared out of control (15, 16). Since 2000, cytokine storm has become increasingly used not only in non-infectious clinical issues but also in diseases caused by cytomegalovirus, influenza virus, variola virus, severe acute respiratory syndrome coronavirus (SARS-CoV), Middle East respiratory syndrome coronavirus (MERS-CoV), and the currently pandemic SARS-CoV-2 (17–19). Recently, cytokine storm is also proved to be associated with the underlying pathogenesis of viral hemorrhagic disease such as Lassa fever, yellow fever, dengue, and Ebola viral disease (20).

Given the fact that ASF and these viral infectious diseases share very similar clinical and pathological features and that ASFV-related elevation of secretion level of several cytokines *in vivo* and *in vitro* was reported (1, 8), a hypothesis could be raised that infection of ASFV in domestic pig could result in a systematic cytokine storm, which may be associated with the viral pathogenesis. However, so far, a global picture of the kinetics of ASFV-induced cytokine release *in vivo* is not available (21). Here, we performed experiments in domestic pigs to analyze the representative circulating interferons (IFNs), interleukins (ILs), growth factors, tumor necrosis factors (TNFs), and chemokines promoted by infection of type II virulent ASFV strain and determined a comprehensive increase of pro-inflammatory cytokines that drew an image of a typical cytokine storm. Furthermore, we also showed that cytokines such as IL-1β, IL-6, TNF-α, IL-8, and IL-10 may act as indicators of a lethal outcome at the endpoint of the infection process.

## Materials and Methods

### Cell Culture and Virus

Bone marrow-derived macrophage (BMDM) was prepared for virus propagation. Briefly, bone marrow was collected by washing the medullary cavity of ribs and tibias obtained from local domestic pigs with RPMI 1640 (Gibco); progenitor cells were collected after lysis of erythrocyte and double rinses with phosphate-buffered saline (PBS). The cell pellets were subsequently resuspended and grown in RPMI 1640 supplemented with 10% fetal bovine serum (Gibco), 100 μg/mL penicillin, 100 μg/mL streptomycin, and 250 ng/mL amphotericin B (Gibco). Cells were seeded at a density of 10^7^ cells/mL in p100 culture dishes (Corning) and cultured in an incubator at 37°C in a 5% CO_2_ atmosphere saturated with water vapor. After a 1-week induction with 20 ng/mL granulocyte-macrophage colony-stimulating factor (R&D, 711-PG), the adherent BMDM cells were either directly employed for virus propagation or harvested *via* trypsin digestion for further use. Clean cells, free of ASFV, classical swine fever virus, parainfluenza virus 5, pig bovine viral diarrhea virus, porcine epidemic diarrhea virus, porcine reproductive and respiratory syndrome virus, pseudorabies virus, porcine parvovirus, porcine circovirus 1/2, and porcine adenovirus, were screened using conventional or quantitative polymerase chain reaction (qPCR) assays (technological details are available upon request).

ASFV SY18 (MH766894.1), a type II isolate firstly obtained from specimens in the initial ASF outbreak in Shenyang, China, 2018, is maintained at −80°C in the biosecurity level 3 lab in the Institute of Military Veterinary Medicine (5). Virus stocks <5 passages were prepared as simply described below. BMDM cells were seeded at a density of 2 × 10^7^ per p100 culture dish and were infected 12 h later with ASFV at a multiplicity of infection of 0.5. At 96 h post infection, the cells were harvested and underwent three freeze–thaw cycles. The supernatant containing the viruses was collected after a centrifugation at 3,000 rpm, 4°C for 15 min, and stored in aliquots at −80°C (22). The viruses were then titrated using the BMDM cells *via* fluorescent focus assay adapted from previous methods (22, 23). The fluorescein isothiocyanate-labeled monoclonal antibody against ASFV early protein P30 involved in this assay was laboratory-made based on conventional procedures (24).

### Animal Trials

For biosafety reasons, animal-involved research upon infection of virulent ASFV was firstly permitted by the Ministry of Agriculture and Rural Affairs of the People's Republic of China. With regard to the animal ethics and welfare, the experimental design was then evaluated and approved by both the Animal Welfare and Ethics Committee of the Institute of Military Veterinary Medicine and the animal biosecurity level 3 (ABSL-3) lab, under reference number JSYABSL3-D-2(2)-JL084-2019.

Due to the rule of reduction, the sample size was restricted to three. To ensure the validity of the conclusion drawn, the animal experiment was permitted to perform twice. In each trial, three 2- to 4-month-old Duroc–Landrace–Yorkshire piglets (*Sus scofa domestic*) with weight between 15 and 25 kg were obtained from a local farm with high biosecurity standards and hygiene. Before being introduced into the laboratory, the healthy piglets were screened *via* conventional or qPCR assays. Those free of the clinically common porcine viruses mentioned in the previous subsection were transferred to the ABSL-3 facilities and were subcutaneously injected with implantable animal electronical microchip. Pigs were supplied with commercial feed twice per day and clean water at all times. After 7 days of acclimatization, the pigs were intramuscularly inoculated at the left hind limb with 1 mL of PBS diluted supernatant containing 10^3^ fluorescence focus unit of ASFV SY18.

Clinical signs were monitored around 9 am every day after the first feed. Rectal temperatures were measured with the values obtained from the electronical microchip (unstable) as reference. Three to four milliliters of blood samples from the precaval vein was collected from each pig daily. Sera were then isolated by centrifugation at 2,000 rpm for 20 min after clotting at room temperature for 2–3 h and were aliquoted and stored at −40°C. Viremia of the pigs was determined by detecting the presence of ASFV genomic DNA in blood through qPCR method (25). The trial was ended by spontaneous death or euthanasia using pentobarbital at the humane endpoint.

### Enzyme-Linked Immunosorbent Assay

Circulating cytokines in serum were quantitatively analyzed *via* sandwich enzyme-linked immunosorbent assay (ELISA). Commercial ELISA kits of IL-1β (PLB00B), IL-6 (P6000B), TNF-α (PTA00), IL-8 (P8000), IL-10 (P1000), IL-12/IL-23 p40 (P1240), and transforming growth factor (TGF)-β1 (MB100B) were obtained from R&D Systems; kits of RANTES (regulated upon activation, normal T cell expressed and secreted) (RAB1881), IP-10 (RAB1132), IFN-α (RAB1131), and IFN-γ (RAB0226) were provided by Sigma-Aldrich®. The ELISA kit targeting porcine IFN-γ (ES9RB) from Invitrogen was also used to further confirm the results of the level of IFN-γ. All detections were performed strictly according to the instructions supplied by the manufacturers. The concentrations of cytokine were calculated according to the standard curve constructed independently in each assay. All assays were performed twice, and in each assay, all samples were tested in duplicate.

### Statistical Analysis

The data were statistically analyzed with GraphPad Prism 8.0 (GraphPad Software Inc., USA). Data normality was tested with the Shapiro–Wilk normality test. The significance of temperature change was analyzed with ordinary one-way analysis of variance (ANOVA). Cytokine levels of each pig were analyzed using two-way ANOVA with Tukey's multiple comparison test. Unless indicated otherwise, data are shown as means with standard deviation (SD) of different measures in figures. Statistical significance was defined as ^*^, *p* < 0.05; ^**^, *p* < 0.01; ^***^, *p* < 0.001; and ^****^, *p* < 0.0001.

## Results

### Clinical Observation

In the first animal trial, pigs were marked as 0#, 5#, and 3#, according to the last number of the microchip code. Among them, pig #3 died at day 5 post inoculation (dpi); pig #0 died spontaneously overnight from 7 to 8 dpi; thereafter, pig #5 was euthanized at 8 dpi since it developed a very similar clinical process as that of pig #0. Pigs used for the other trial were remarkably smaller with average body weight of 18 kg and were designated as #4, #7, and #8. Pig #8 was defeated by ASFV infection at 7 dpi. The next day, we humanely sacrificed pig #7 because of a rather coincident clinical manifestation as that of pig #8. However, pig #4 presented only mild clinical signs until 12 dpi and was euthanized at 14 dpi when it developed severe disease. Given the fact that pigs generally died from SY18 infection during 7–9 dpi in our preliminary experiments, only pigs #0, #5, #7, and #8, which showed typical disease courses, were included for data analysis due to the concern of statistical bias considering the very limited sample size.

**Figure 1:**
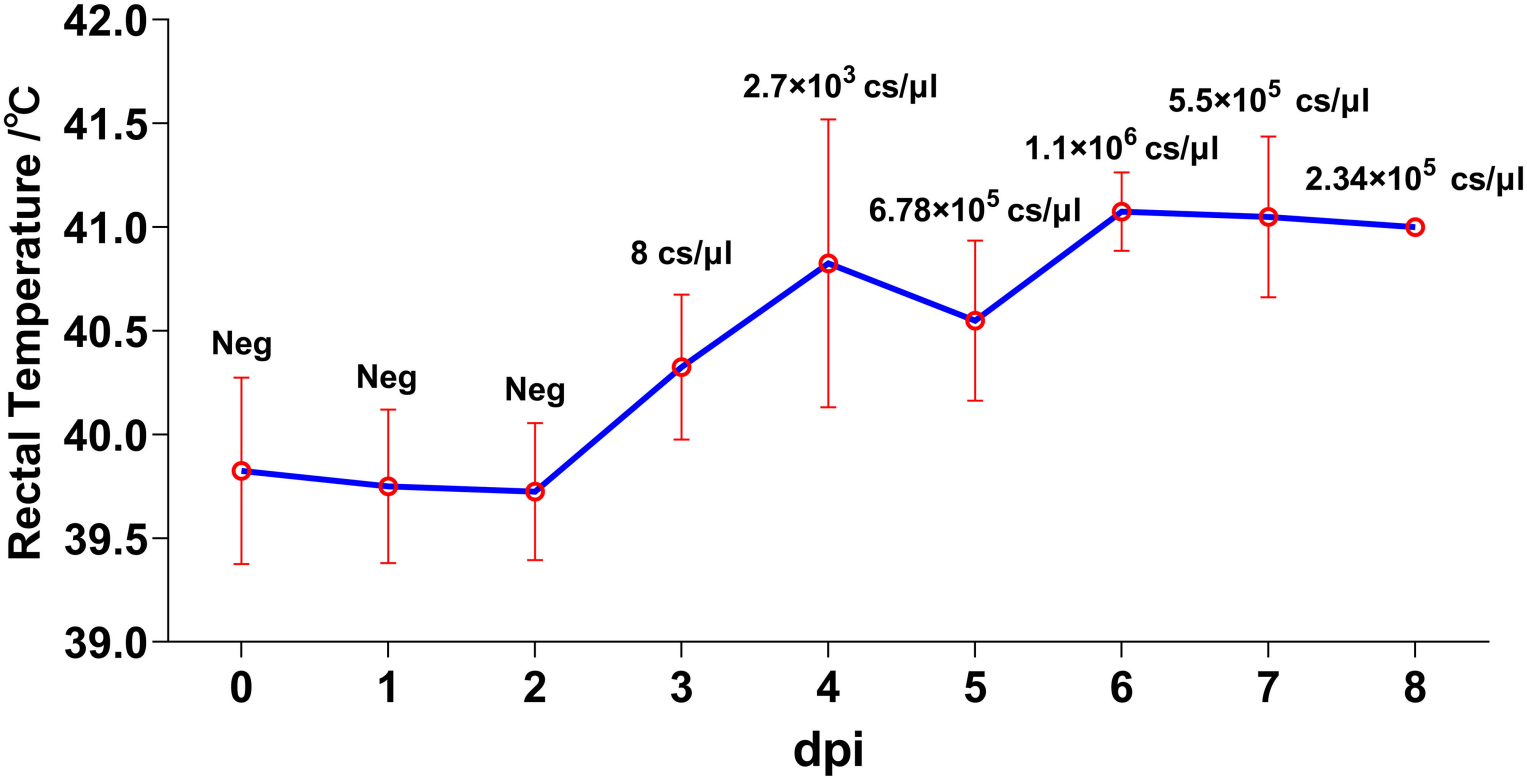
Kinetics of rectal temperatures. Animals were inoculated with 1,000 FFU (1 mL) of African swine fever virus (ASFV) SY18 intramuscularly. Rectal temperature and clinical signs were monitored daily. Data are shown as mean (SD). Viremia was analyzed through quantitative PCR (qPCR) method. The virus titers in blood were quantified by calculating the concentrations of ASFV genome copies *via* a standard curve based on serial diluted plasmids bearing the B646L gene. Values (copies/μL) are shown above the points.

Despite different ages, pigs in both trials developed similar disease courses. The most common clinical feature, fever, could be generally observed since 3 dpi and continued until the termination except for pig #7, with onset of this clinical sign at 5 dpi (Figure 1). Following the initiation of fever, depression and feed intake reduction were observed. In the second half of the disease progression, huddling and incoordination occurred in all animals. Viremia could be detected in accordance with the start of fever at a relatively low level at 3 dpi with a genome concentration of approximately 8 copies/μL, which rose rapidly in 2 days to 6.78 × 10^5^ copies/μL in average and remained at high levels from 5.5 × 10^5^ to 2.34 × 10^6^ copies/μL (Figure 1).

### ASFV Infection Induced a High Level of Type I IFN Secretion

**Figure 2:**
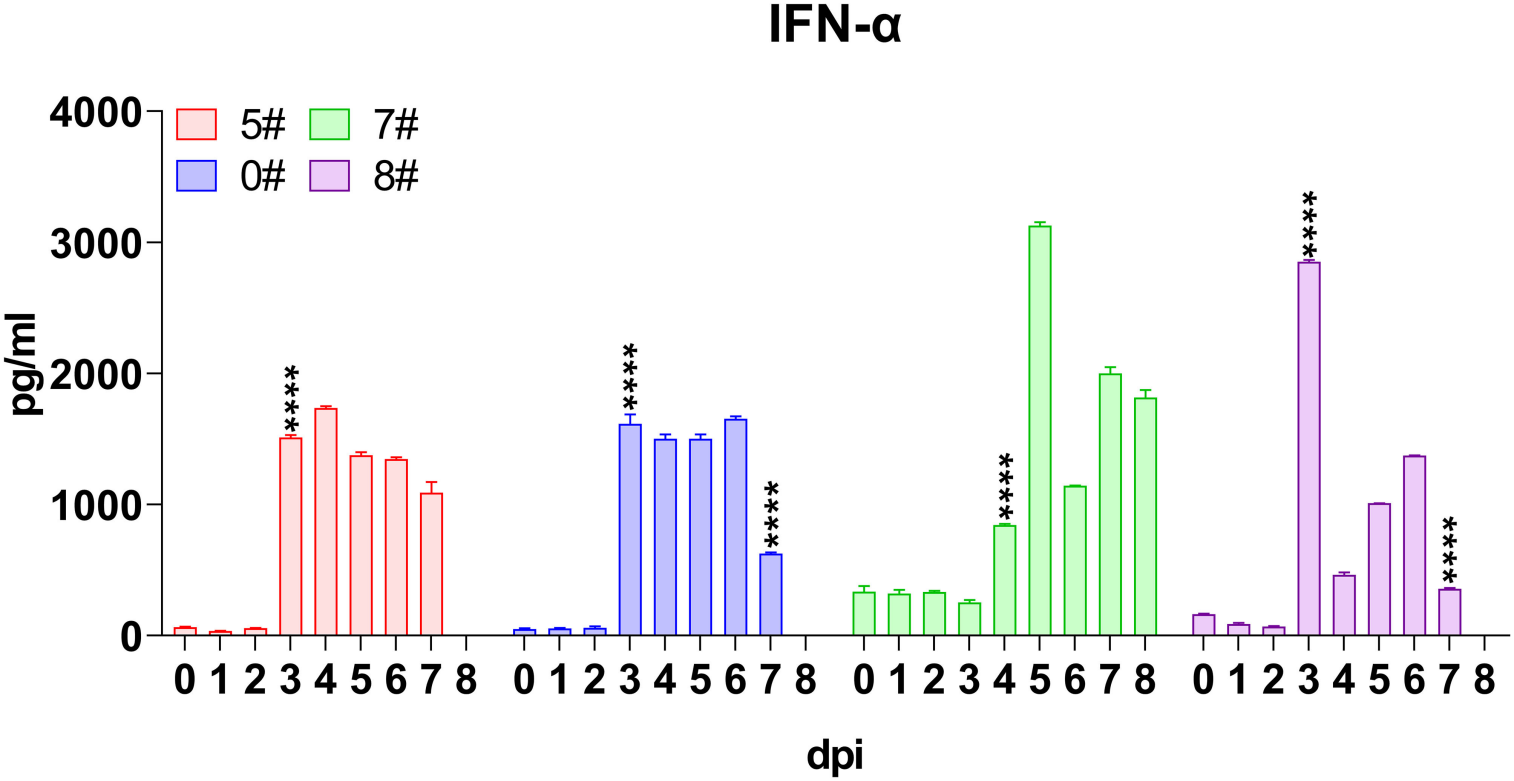
Kinetics of serum interferons. Sera were collected daily both before and after virus inoculation. The concentrations of interferon (IFN)-α in each pig included for analysis were measured *via* quantitative ELISA. IFN-γ was undetectable and thus not shown. Data of different individuals are distinguished by color and are shown as mean (SD). Asterisks indicate statistical significance.

IFNs are widely accepted as central factors antagonizing virus infection, among which type I IFNs, including IFN-α and IFN-β, play critical roles in innate immune response (26). Therefore, we firstly analyzed the level of circulating IFN-α in pigs infected by SY18. In the first trial, IFN-α in both pigs exploded at 3 dpi, with a steep increase of average level from 57 to 1,570 pg/mL and remained at high levels (Figure 2). In parallel, despite a higher basal level, IFN-α in sera of pigs #7 and #8 still displayed a process of dramatic rise of 20–40 times to apex at 5 and 3 dpi, but a decrease of several times was observed thereafter (Figure 2). Interestingly, in pigs #0 and #8, which spontaneously died, the IFN-α level significantly reduced for about three times at the terminal, which indicated a checkpoint of the disease process.

In contrast, the only member of type II IFN, namely IFN-γ, which largely represented the intensity of adaptive immune response was undetectable all through the infection, although two distinct commercial ELISA kits were used to validate each other.

### Pro-inflammatory TNF and ILs Were Comprehensively Elevated Upon Virulent ASFV Infection

**Figure 3:**
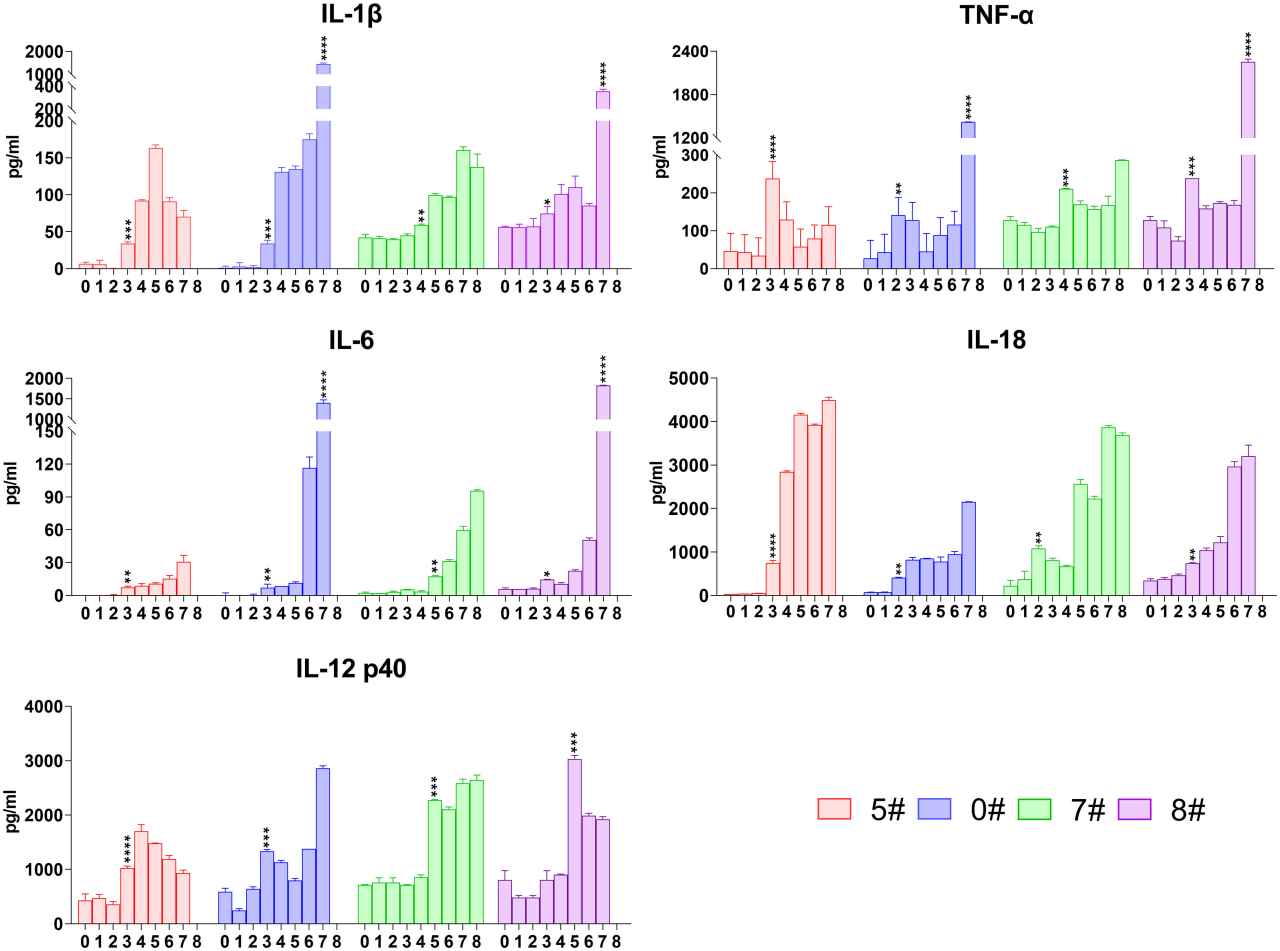
Kinetics of serum representative pro-inflammatory cytokines. The concentrations of interleukin (IL)-1β, tumor necrosis factor (TNF)-α, IL-6, IL-18, and IL-12 in each pig were detected *via* quantitative ELISA. Data of different individuals are distinguished by color and are shown as mean (SD). Asterisks indicate statistical significance.

We subsequently analyzed the kinetics of the most intensive pro-inflammatory cytokines including TNF-α, IL-1β, IL-6, and IL-18 in sera of pigs upon SY18 infection. IL-1β became increased since 3 or 4 dpi but showed slightly different patterns in the two trials. IL-1β levels of pigs #5 and #0 increased nearly 10 times of the basal level from 3 to 34 pg/mL at 3 dpi, whereas that of pigs #7 and #8 displayed a relatively gentle elevation (Figure 3). In parallel with the pattern observed in IFN-α kinetics, there was a sharp increase of about 4–10 times in 24 h of IL-1β in pigs #0 and #8 just before the spontaneous death (from 175 to 1,450 pg/mL and from 85 to 356 pg/mL) (Figure 3). With regard to TNF-α, the level started to rise at 2–4 dpi with a relatively fierce manner (from 65 to 208 pg/mL in average), but declined rapidly thereafter, and became increased again (Figure 3). A drastic increase in the terminal phase in pigs #0 and #8 was also observed (from 115.6 to 1,419.4 pg/mL and from 167.9 to 2,252.3 pg/mL) (Figure 3). The synthesis of IL-6 increased with average level from 2.4 to 11.6 pg/mL at 3 or 5 dpi and kept rising throughout the disease. Again, the concentration in pigs #0 and #8 promptly rose from 116.5 to 1,403.4 pg/mL and from 50.5 to 1,824.3 pg/mL, respectively, during the last 24 h before death (Figure 3). IL-18 is another member of IL-1 cytokine family and plays a protective role in many virus infections (27). Results showed that ASFV infection induced a rise of the level of IL-18 very early at 2 or 3 dpi (from 236.2 to 739.2 pg/mL on average), and this increase tendency remained throughout the disease (Figure 3).

For technical convenience, we directly tested the level of IL-12 p40 that is synthesized after stimulation but not constitutively expressed as p35 to represent the level of IL-12 induced by the intracellularly infected SY18. Like other pro-inflammatory cytokines, secretion of IL-12 also became increased at 3 or 5 dpi (from 688.2 to 1,915.4 pg/mL on average) and maintained at high level until terminals (Figure 3).

### Multiple Chemokines Were Involved in the Response Against ASFV Infection

**Figure 4:**
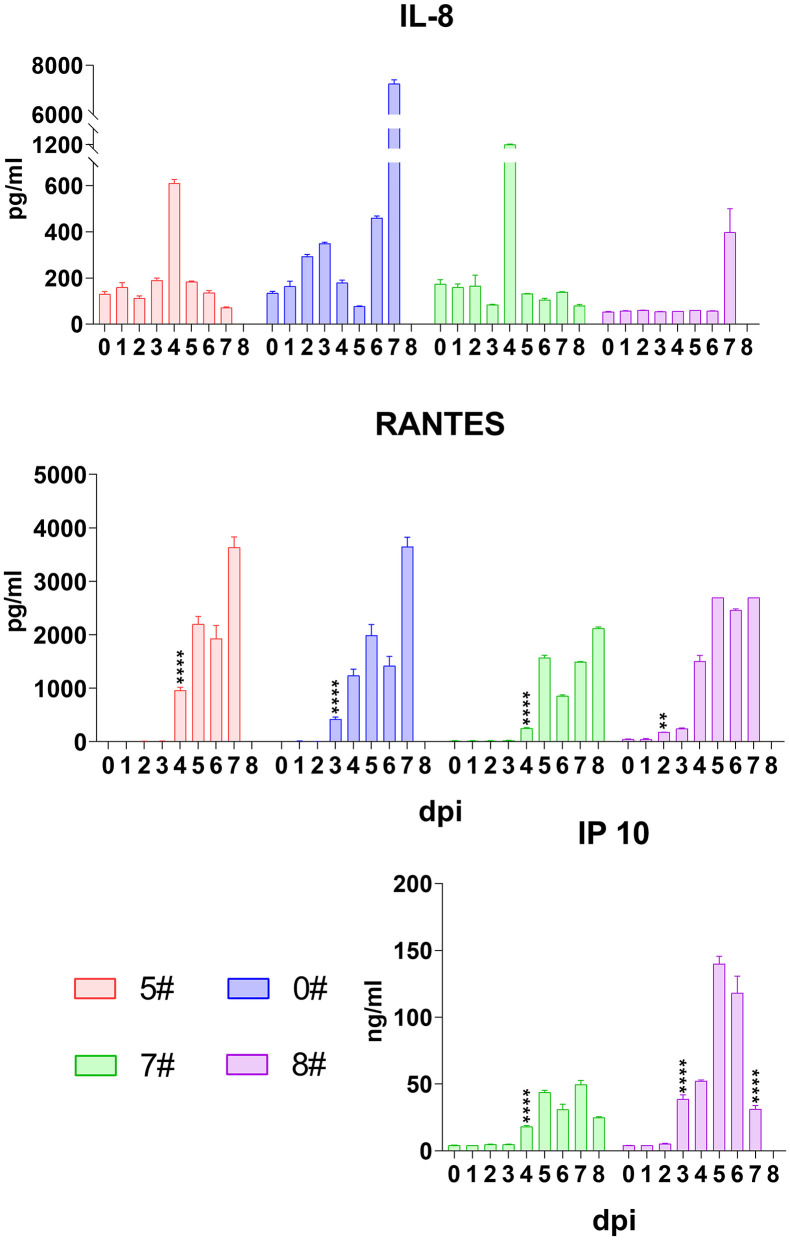
Kinetics of serum chemokines. The serum levels of interleukin (IL)-8, RANTES (regulated upon activation, normal T cell expressed and secreted), and interferon (IFN)-γ-induced protein 10 (IP-10) in each pig were evaluated *via* quantitative ELISA. Data of different individuals are distinguished by color and are shown as mean (SD). Asterisks indicate statistical significance.

Although neutrophils are normally not involved in virus infection, IL-8 secretion is reported in cytokine storm occurring in viral disease (18, 20). We therefore analyzed the level of this cytokine in pigs upon SY18 infection. Not surprisingly, IL-8 increased, however, following a unique pattern that the level in most animals sharply rose and quickly declined at a short time interval (Figure 4), indicating that the mechanism recovering from IL-8 accumulation of the host functioned at the first half of infection. However, echoing with IL-1β, IL-6, TNF-α, and IFN-α at the final phase, the level of IL-8 in pigs #0 and #8 exploded (from 460.5 to 7,254.7 pg/mL and from 57 to 397.8 pg/mL) at the terminal before spontaneous death (Figure 4), which suggested that IL-8 could be another indicative target of disease progression.

We also analyzed the kinetics of RANTES upon SY18 infection. Results showed that a comprehensive dramatic increase of RANTES initiated at 2–4 dpi and was sustained at high levels in all animals throughout the infection (Figure 4).

Despite a wide range of cell sources, IFN-γ-induced protein 10 (IP-10) is produced, in a greatest proportion, by activated monocytes/macrophages with response to a variable of stimuli, such as human immunodeficiency virus, dengue virus, and hepatitis B virus, and is associated with disease severity (28). In this regard, we also analyzed the secretion of IP-10 during ASFV infection in the second trial. As expected, the serum level of this chemokine dramatically began to increase at 3 or 4 dpi from 5.1 to 28.3 ng/mL on average and kept increasing until the final phase when it declined, showing a similar pattern with IFN-α (Figure 4).

### The Anti-inflammatory Cytokines Were Absent in Neutralizing the Prolonged Pro-inflammatory Response Upon Infection of ASFV

**Figure 5:**
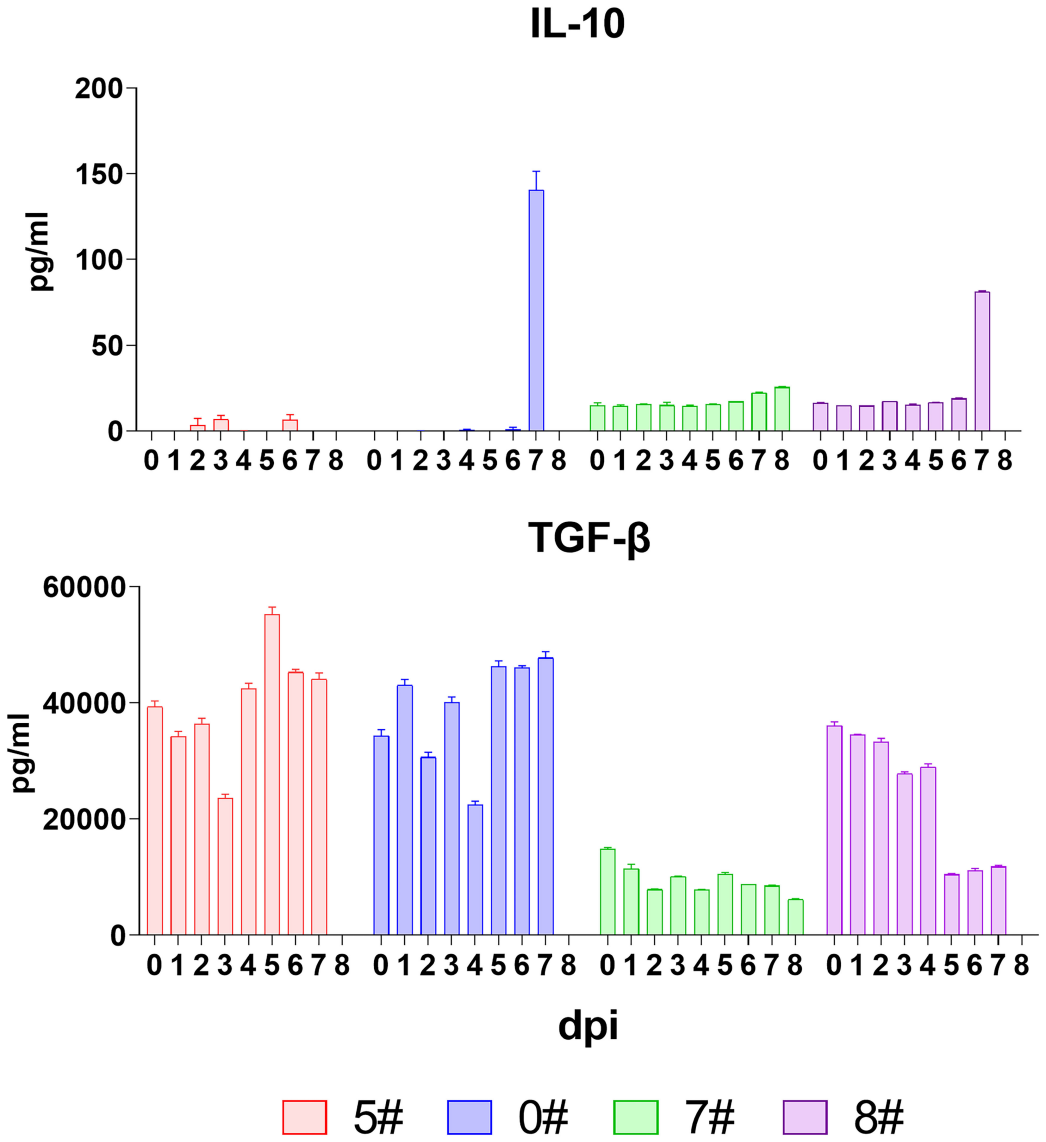
Kinetics of anti-inflammatory cytokines. The serum levels of interleukin (IL)-10 and transforming growth factor (TGF-β) in each pig were evaluated *via* quantitative ELISA. Data of different individuals are distinguished by color and are shown as mean (SD).

The anti-inflammatory cytokines exert a negative feedback regulation loop to balance the host inflammatory response during virus insults and thereby prevent detrimental immunopathology induced by excessive pro-inflammatory cytokine secretion and exuberant immune cell activation (29, 30). Among the anti-inflammatory cytokines, IL-10 plays a non-redundant role *in vivo*, and thus, we measured the serum level of this cytokine (31–33). Results showed that IL-10 was absent in the early host response against ASFV infection, but the serum level of IL-10 in pigs #0 and #8 drastically increased (from 1.1 to 140.5 pg/mL and from 18.9 to 81.2 pg/mL) at the final phase before spontaneous death (Figure 5). TGF-β, another important immunoregulatory cytokine, was also analyzed. Although this cytokine has a variety of cell sources, we did not detect a consistent increase but sometimes a decrease instead (Figure 5). These collectively indicated that the mechanism balancing the pro- and anti-inflammatory response was interrupted during SY18 infection.

## Discussion

The major focus of our study was to characterize the kinetics of cytokines secreted *in vivo*, clarifying whether a cytokine storm was involved in the pathogenesis of ASFV. Monocyte-macrophages, the primary target cells for replication of ASFV, play a central role in cytokine network. Thus, several *in vitro* studies involved the comparison of cytokine response of macrophage upon infection of ASFV with different virulence and found that low-virulent ASFV NH/P68 induced increased transcription of IFN-α, IL-6, TNF-α, and IL-12 in comparison with the virulent ASFV L60 (34, 35) and that low virulence ASFV OURT88/3 or avirulent BA71V induced higher expression of IFN-β, IL-18, IL-1β, IL-1α, CCL4, IL-8, and IP-10 compared to the virulent ASFV Benin 97/1 or 22653/14 (36, 37). However, results from *in vitro* and *in vivo* studies were somewhat discrepant. It was demonstrated that TNF-α and IFN-α/β could be readily detected in the serum of animals infected with virulent isolates (38, 39), which was further confirmed by recent studies (40, 41). Our work expanded the concept of knowledge about circulating cytokines induced by virulent type II ASFV in domestic pigs. The virulent ASFV SY18 induced continuous increase of almost all key pro-inflammatory cytokines involved in virus infection but not the anti-inflammatory cytokines, IL-10 and TGF-β1, which, at the first time, clearly drew an image of a cytokine storm.

The clinical forms of ASFV infection include peracute, acute, subacute, and chronic, while a virulent type II field strain generally induces acute infection (8). According to the pattern of kinetics of cytokine release in the present and previous studies (41), the disease course of an acute infection with highly virulent ASFV could be roughly divided into three phases: the primary phase, covering the period of 0 to 2 dpi with no change of the serum cytokine levels and no clinical symptoms; the clinical phase, displaying progressive clinical features, extensively upregulated pro-inflammatory cytokine release including TNF-α, IFN-α, IL-1β, IL-6, IL-8, IL-12, IL-18, RANTES, and IP-10, and sustained fever from 3 to 7 dpi; and the terminal phase, a constricted interval basically from 7 to 8 dpi characterized with an additional sharp increase of multiple cytokines (TNF-α, IL-1β, IL-6, IL-8, and IL-10) but a relatively recovered IFN-α.

Despite essential roles of cytokines in responding appropriately to infectious insults and controlling the invading microbes, an excessive and prolonged secretion of pro-inflammatory cytokines is closely related to immunopathological outcomes (15). For instance, overexpressed TNF-α, IL-1, and IL-6 could induce fever, general malaise, fatigue, vascular leakage, diarrhea, cardiomyopathy, lung injury, vascular leakage, acute-phase protein synthesis, complement system activation, and diffuse intravascular coagulation (15, 42). In addition, sustained and excessive IFN-α/β could also result in a detrimental response (26). This was in line with the case in the current study in which a comprehensive elevation of pro-inflammatory cytokines was observed accompanied by severe clinical manifestations.

During the clinical phase, the increased viremia showed that the virus began to efficiently spread through circulating system, accompanied by the first wave of pro-inflammatory cytokine release. The uncontrolled and sustained increase of cytokines could possibly result from multiple factors. Accumulating stimulation by newly synthesized ASFV components and positive feedback of the pro-inflammatory cytokines through auto-, para-, and endocrine pathways might contribute to the excessive activation of both infected and non-infected macrophages, leading to amplifying cytokine secretion (26). IL-1β, IL-6, and TNF-α are largely early products of immune response against invasive microbes. After activation, IL-1β and TNF-α can further act as activators of bystander cells for IL-6 secretion, which can in turn enhance the activity of IL-1β and TNF-α, magnifying the inflammatory response (43, 44). On the other hand, the silence of anti-inflammatory cytokines exacerbated the imbalance of immune homeostasis (14, 33). Although the prototypical anti-inflammatory cytokine, IL-10, is frequently involved in cytokine storms induced by T cell engaging immunotherapy, influenza virus and dengue virus infection (15, 18, 20), we did not detect any secretion of this regulatory cytokine in pigs upon ASFV SY18 infection except for the final phase, indicating that IL-10 was prevented or left out in controlling the ASFV-derived immunopathology. IL-10 is mainly produced by M2 polarized macrophages, myeloid dendritic cells, T helper 2 cells (Th2), and, at certain conditions, Th1, Th17, Th9, regulatory T cells (Treg), B cells, and mast cells (33). Although it was reported that IL-10 could show an IFN-γ-mediating pro-inflammatory effect in the presence of an ongoing serious inflammatory process (45), the IFN-γ secretion was not detected in the pigs in the current study, and therefore, the increased level of IL-10 observed at the endpoint might more likely suggest a desperate struggle of host against the inflammatory impact. In this point of view, IL-10 seems to be an indicative hallmark of an irrecoverable status, rather than one of the causes, of cytokine storm during acute ASF. This is in agreement with the recent observations that an elevation of IL-10 was likely to be related to a lethal outcome (37, 46, 47).

The type I IFNs have been shown to be indispensable in elimination of invading virus that otherwise will be pathogenic. To survive the host's immune system, most viruses have evolved multiple strategies to counteract the type I IFN response (48). In the case of ASFV, MGF360-15R, MGF505-7R, pI329L, pA238L, and pDP96R were reported to suppress the type I IFN induction as well as NF-κB-dependent pro-inflammatory cytokine secretion *via* various mechanisms (49–52). This could be a substantial cause of the unchanged level of type I IFN as well as other pro-inflammatory cytokines during the primary phase of acute ASF. Due to the incapability of type I IFN induction by virulent ASFV in macrophages (34, 37, 51), the dramatical elevation of IFN-α during the clinical phase of infection could be a result of secondary activation of plasmacytoid dendritic cells (53). However, as shown in the studies based on SARS-CoV (17, 54), the postponement of type I IFN secretion accompanied by robust virus replication could promote the accumulation of activated monocyte-macrophages, resulting in raised cytokine levels and impaired T cell responses.

The impairment of T cell response upon infection of virulent ASFV was recently reported (47, 55). This is in line with our study in which IFN-γ was proved to be absent throughout the ASFV SY18 infection. The influence of IFN-γ response in ASFV infection remains controversial. While the correlation of IFN-γ response with protection against ASFV were reported in some studies (40, 56, 57), other data did not support this relativity (58, 59). On the other hand, the absence of IFN-γ might also reflect an impaired activation of natural killer (NK) cell, which was another major producer of this cytokine and played a crucial role in counteracting virus infection (60, 61). This is in accordance with the previous study in which the NK cell activity negatively correlated with the ASFV virulence and animal clinical manifestations (62). Collectively from these results and our data, IFN-γ may play an essential but insufficient role in combating ASFV infection. Nevertheless, virulent ASFV could block IFN-γ responses of both NK cells and T cells.

Chemokines play crucial roles in leukocyte movement and act in concert with other cytokines to manage tissue infiltration and inflammation (63). Due to these fundamental functions, chemokines are often found to contribute to occurrence of cytokine storm (18, 20). Reports involving the *in vivo* roles of chemokines upon ASFV infection are limited. Zhang et al. (64) described a mild upregulation (1.4 ×) of RANTES in macrophages upon infection of virulent ASFV at an early stage (4 h post infection), whereas the same group also reported that at late stage, the mRNA levels of RANTES remained stable following infections with either attenuated or virulent ASFV (65). Moreover, the secretion of IL-8 by macrophage was even decreased upon infection of virulent ASFV (65). Our study showed that secretions of IL-8, RANTES, and IP-10 by domestic pigs upon infection of virulent SY18 all increased during both the clinical and final phases. This is in agreement with a previous study in which a continuous increase of IL-8 in pigs upon infection of virulent ASFV was described (41). This obvious discrepancy of chemokine secretion between *in vitro* and *in vivo* might indicate non-macrophage sources of these inflammatory mediators in pigs infected by virulent ASFV.

Taken together, the prototypical isolate ASFV SY18 that was primarily derived from specimens collected from the first outbreak of ASF in China induced acute ASF in domestic pigs. A representative disease course could be divided into three periods, the primary phase, the clinical phase, and the terminal phase, which were immune-silent, dysregulated, and uncontrolled, respectively. Accompanied by the onset of severe clinical signs and viremia, a cytokine storm occurred characterized by a tendency of significantly increased and sustained secretion of TNF-α, IFN-α, IL-1β, IL-6, IL-8, IL-12, IL-18, RANTES, and IP-10. Moreover, secondary drastic increase of the levels of TNF-α, IL-1β, IL-6, and IL-8, as well as elevated IL-10, could be hallmarks of an irreversible immune status, foreboding a lethal outcome. Although the molecular mechanisms of cytokine storm are far from understood, it seems that the ASFV-caused cytokine storm arises from the combination of delayed innate response in the primary phase, the suppressed anti-inflammatory response, and the impaired T cell response in the clinical phase. However, the underlying molecular mechanisms require further investigations.

Although our study has limitations that the blood situation may not directly indicate the exact reaction at the loci where viruses replicate and that the restricted number of animals used could affect the data precision, the conclusions drawn from the two independent trials as well as another experiment in which animals were inoculated *via* oral routes (data not shown) all indicated a correlation between clinical signs and an excessive pro-inflammatory response, which paved a way for further understanding of the molecular basis of ASFV pathogenesis.

## Data Availability Statement

The original contributions presented in the study are included in the article/supplementary materials, further inquiries can be directed to the corresponding author/s.

## Ethics Statement

The animal study was reviewed and approved by the Animal Welfare and Ethics Committee of the Institute of Military Veterinary Medicine.

## Author Contributions

SW, SZ, and RH conducted the whole project. SW, JZ, YZ, LW, JY, YQ, XZ, and FM performed the animal experiment and collected the primary data. SW, TC, YW, and FZ verified and analyzed the data. SW and SZ drafted the manuscript. All authors read and approved the final version of the manuscript.

## Conflict of Interest

The authors declare that the research was conducted in the absence of any commercial or financial relationships that could be construed as a potential conflict of interest.
